# Tolosa–Hunt Syndrome: A Painful Ophthalmoplegia

**DOI:** 10.1155/2020/8883983

**Published:** 2020-11-07

**Authors:** Marcellin Bugeme, Ousmane Cissé, Olivier Mukuku, Amadou Gallo Diop

**Affiliations:** ^1^Faculty of Medicine, University of Lubumbashi, Lubumbashi, Democratic Republic of the Congo; ^2^Université Cheikh Anta Diop, Dakar, Senegal; ^3^Institut Supérieur des Techniques Médicales de Lubumbashi, Lubumbashi, Democratic Republic of the Congo

## Abstract

Tolosa–Hunt syndrome is a painful ophthalmoplegia characterized by recurrent unilateral orbital pain, ipsilateral oculomotor paralysis, and a rapid response to steroids. Our report describes a 37-year-old young woman who presented with right ptosis, ipsilateral ophthalmoplegia, and painful headache with no other neurological deficits in which all biological and neuroimaging investigations were normal. Complete recovery within one week of corticosteroid therapy was observed. This is probably the first case of Tolosa–Hunt syndrome reported in Dakar, Senegal.

## 1. Introduction

Tolosa–Hunt Syndrome (THS) is a rare neurological condition of unknown etiopathogenesis, characterized by unilateral pain and ipsilateral oculomotor paralysis [1]. This disease is caused by an unspecific inflammation (of unknown origin) in the region of the cavernous sinus and the sphenoid cleft. Its evolution is considerably shortened by corticosteroid therapy correctly within 72 hours [2]. In 1954, Tolosa reported the first patient with this syndrome, who presented with left orbital pain, ipsilateral progressive visual loss, total left ophthalmoplegia, and reduced sensation over the first division of the trigeminal nerve. After 7 years of this, in 1961, Hunt described this clinical entity, on the basis of six patients [3].

We report a case of a patient admitted with a right droopy eyelid and right orbital headache evolving for two months before her consultation. Neurological examination revealed a right ptosis and an ipsilateral oculomotor paralysis. The additional examinations performed had returned to normal. After a week of corticosteroid therapy, all the symptoms had completely subsided.

## 2. Case Report

A 37-year-old Senegalese woman with hypertension history, presented with right, excruciating, permanent, intense, nonpulsatile orbital headache associated with right ptosis without visual loss. The onset of symptoms dated back to two months before her arrival at the hospital with headache that had appeared about 11 days before a right drooping eyelid, associated with ipsilateral orbital pain. The patient had not yet received any treatment.

Medical examination showed the patient in good general state, the mucosa were well colored, there were no folds of dehydration or malnutrition, no edema of the lower limbs, and the calves were flexible. The vital parameters were within the norms.

**Figure 1:**
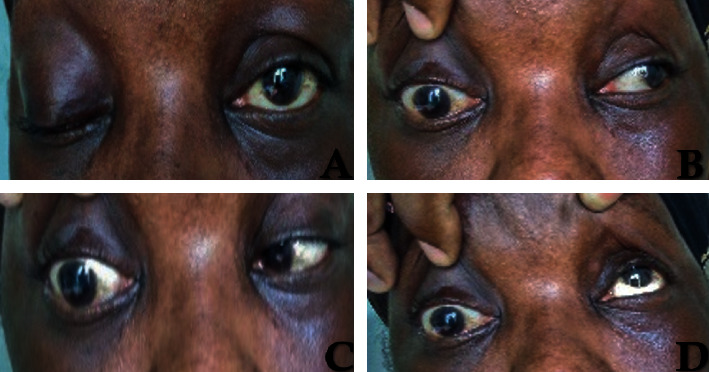
(a) Right ptosis. (b) Paresis of the third cranial nerve. (c) Paresis of the sixth cranial nerve. (d) Paresis of the fourth cranial nerve.

**Figure 2:**
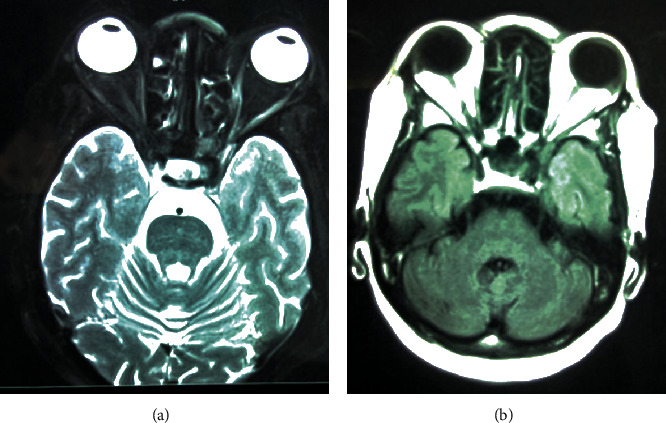
MRI axial slice of the brain in (a) T2 sequence and (b) T2-FLAIR sequence after contrast injection showing no abnormalities in the cavernous sinus.

Neurological examination found a paralysis of the right oculomotor nerves (III, IV, and VI) associated with a homolateral ptosis (Figure 1). The rest of the exam was unremarkable. Brain magnetic resonance imaging (MRI) with contrast was performed and was unremarkable (Figure 2).

Laboratory investigations including complete blood count, erythrocyte sedimentation rate, fasting blood sugar, renal and liver functions, angiotensin-converting enzyme, antinuclear antibody, serum protein electrophoresis, antineutrophil cytoplasmic antibody, C-reactive protein, and viral serologies (hepatitis A, B, and C, human immunodeficiency virus, and venereal disease research laboratory) testing were either normal or negative. The cerebrospinal fluid analysis was within normal ranges with normal glucose and protein and negative cytology. Intradermal tuberculin test was negative.

**Figure 3:**
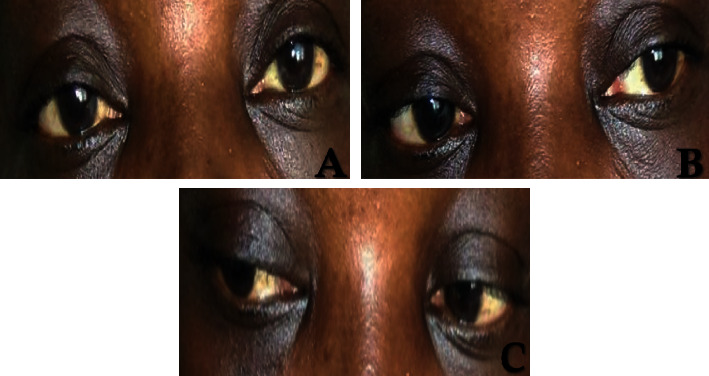
(a) Regression of right ptosis. (b) Complete remission of the third cranial nerve. (c) Complete remission of the sixth cranial nerve.

The patient had received a treatment with oral corticosteroids (prednisone 60 mg daily) combined with adjuvant therapy (calcium 500 mg per day orally and potassium 600 mg per day orally). After a week of treatment, complete remission of oculomotor disorders and other symptoms was noted (Figure 3).

The probable diagnosis of Tolosa–Hunt Syndrome was retained in the face of the negativity of all investigations carried out as well as spectacular response to treatment.

## 3. Discussion

Tolosa–Hunt syndrome is a painful ophthalmoplegia due to nonspecific inflammation of the cavernous sinus. Existence of this syndrome has long been debated, and its nosological framework was discussed [2].

Pathophysiological and etiopathogenic mechanisms of THS remain controversial. It is a diagnosis of exclusion retained after eliminating other etiologies of painful ophthalmoplegia [4, 5]. Following essentially clinical criteria has been established by Hunt [5, 6]: one or more episodes of unilateral orbital pain persisting for weeks without treatment; paresis of the third, fourth, and/or sixth cranial nerve and/or detection of granulomas by MRI or by biopsy; paresis in conjunction with sudden onset of eye pain or pain within 2 weeks of ocular dysfunction; pain and paresis disappear within 72 hours if treatment with corticosteroids is adequate; other etiologies have been excluded by appropriate investigations.

Age of patients varies between 4 and 75 years [7], but THS can affect people of any age, without predominance of sex. In general, it is unilateral, but cases of bilaterality have been reported [8]. Our patient was 38 years old, and she presented with unilateral symptoms. The diagnosis of this syndrome was retained in the face of the negativity of all the investigations carried out as well as the response to treatment. Extensive diagnosis tests to exclude other common etiologies of painful ophthalmoplegia were not found in our patient.

Many studies have looked at THS and the contribution of MRI, an examination essential for the diagnosis of this syndrome, because it allows [9] to eliminate a specific inflammatory process (sarcoidosis) or tumor and to show the existence of an isosignal zone in T1 and T2 and the deformation of cavernous sinus which are suggestive signs. However, it seems possible that MRI techniques can be faulted and do not detect minimal infiltrates (as is certainly the case with our patient). Failure to visualize these infiltrates by MRI would indicate an early stage of the disease and therefore a better response to treatment [10].

Recently, diagnostic criteria for THS have been published by the International Headache Society in the 3^rd^ edition of International Classification of Headache Disorders (ICHD-3) which places the THS in position 13.8 [11]. This ICHD-3 classification reinforces the important role of MRI in the diagnosis of THS. MRI also allows the exclusion of other similar causes such as lymphoma, meningioma, and sarcoidosis [12]. The MRI was normal in our patient, and patients with THS with normal MRI have already been reported in the literature [2, 12–14]. In addition, in this category, the use of biopsy is considered to highlight a granulomatous inflammation of the cavernous sinus, superior orbital fissure, or orbit [2]. In our context, where optimal conditions are not easily met, a biopsy of a patient suspected of THS with an excellent response to corticosteroid therapy to confirm the diagnosis should not be necessary because it carries a risk of complications. Thus, exposing patients to a risky procedure such as a biopsy should not be a necessity for diagnosis. Kóbor et al. [13] recommend a repeat of the MRI in a patient with an initially normal MRI to reveal new inflammatory signs which could appear months after the initial presentation. Mainly because of costs, our patient did not have MRI in the follow-up, and she was monitored by clinical visits only. The lack of follow-up MRI in our case reflects the day-to-day reality in our settings. Health insurance systems are almost nonexistent, and the population has to take care of itself for medical care.

Zhang et al. [2] proposed introduction of categories of definitive, probable, and possible since it is highly doubtful whether the diagnostic criteria for ICHD-3 are so completely based on it (or on a biopsy). Patients with normal MRI or with a time interval between the onset of pain and ophthalmoplegia of more than 2 weeks or whose diagnosis is dependent on pain and paresis resolving within 72 hours of starting corticosteroid therapy cannot be diagnosed as definitive TSH and is not better explained by another ICHD-3 diagnosis. This is how probable or possible THS are working alternatives. We believe this is reasonable and may be helpful in reducing the financial burden and complications of unnecessary investigations to confirm the diagnosis of THS. In addition, in agreement with Zhang et al. [2], we also believe that the response to steroids is probably still important to support the diagnosis of THS in clinical practice, in the absence of other specific and reliable criteria.

The spectacular efficacy of corticosteroid therapy is suggestive but not specific. Hence it is necessary to conduct prolonged monitoring of several months to retain this diagnosis definitively. Treatment consists of a high dose of oral prednisone for 4 weeks. Significant improvement is often evident from the first 24 hours of treatment [12]. Our patient received a dose of prednisone 60 mg per day, and after a week, a complete dramatic regression of the symptoms was observed. After more than six months of monitoring, no clinical abnormality was observed in our patient. Zhang et al. [2] reported that 77.5% of patients obtained complete relief of orbital pain within a week after the start of steroid treatment. This spectacular efficacy of corticosteroid therapy has been observed in several other studies [3, 5, 15, 16].

## 4. Conclusion

Tolosa–Hunt syndrome is a rare entity well-defined clinically but of unknown etiopathogenesis. It remains a diagnosis of exclusion. In our case, the negativity of investigations and spectacular response to corticosteroids allowed us to retain the diagnosis.

## Data Availability

The data used to support the findings of this study are available from the corresponding author upon request.
